# A Modified BFGS Formula Using a Trust Region Model for Nonsmooth Convex Minimizations

**DOI:** 10.1371/journal.pone.0140606

**Published:** 2015-10-26

**Authors:** Zengru Cui, Gonglin Yuan, Zhou Sheng, Wenjie Liu, Xiaoliang Wang, Xiabin Duan

**Affiliations:** 1 Guangxi Colleges and Universities Key Laboratory of Mathematics and Its Applications, College of Mathematics and Information Science, Guangxi University, Nanning, Guangxi 530004, China; 2 School of Computer and Software, Nanjing University of Information Science & Technology, Nanjing 210044, China; 3 Jiangsu Engineering Center of Network Monitoring, Nanjing University of Information Science & Technology, Nanjing 210044, China; Beijing University of Posts and Telecommunications, CHINA

## Abstract

This paper proposes a modified BFGS formula using a trust region model for solving nonsmooth convex minimizations by using the Moreau-Yosida regularization (smoothing) approach and a new secant equation with a BFGS update formula. Our algorithm uses the function value information and gradient value information to compute the Hessian. The Hessian matrix is updated by the BFGS formula rather than using second-order information of the function, thus decreasing the workload and time involved in the computation. Under suitable conditions, the algorithm converges globally to an optimal solution. Numerical results show that this algorithm can successfully solve nonsmooth unconstrained convex problems.

## Introduction

Consider the following convex problem:

$$\begin{array}{c} \underset{x\in R^{n}}{\text{min}}f\left(x\right), \end{array}$$ (1)

where *f* : ℝ^*n*^ → ℝ is a possibly nonsmooth convex function. In general, this problem has been well studied for several decades when *f* is continuously differentiable, and a number of different methods have been developed for its solution Eq (1) (for example, numerical optimization method [1–3] etc, heuristic algorithm [4–6] etc). However, when *f* is a nondifferentiable function, the difficulty of solving this problem increases. Recently, such problems have arisen in many medical, image restoration and optimal control applications (see [7–13] etc). Some authors have previously studied nonsmooth convex problems (see [14–18] etc).

Let *F* : ℝ^*n*^ → ℝ be the so-called Moreau-Yosida regularization of *f*, which is defined by

$$\begin{array}{c} F\left(x\right):=\min_{z\in R^{n}}\{f\left(z\right)+\frac{1}{2\lambda }{∥z-x∥}^{2}\}, \end{array}$$ (2)

where *λ* is a positive parameter and ‖ ⋅ ‖ denotes the Euclidean norm. The problem Eq (1) is equivalent to the following problem

$$\begin{array}{c} \min_{x\in R^{n}}F\left(x\right). \end{array}$$ (3)

It is well known that the problems Eqs (1) and (3) of the solution sets are the same. As we know, one of the most effective methods for problems Eq (3) is the trust region method.

The trust region method plays an important role in the area of nonlinear optimization, and it has been proven to be a very efficient method. Levenberg [19] and Marquardt [20] first applied this method to nonlinear least-squares problems, and Powell [21] established a convergence result for this method for unconstrained problems. Fletcher [22] first proposed a trust region method for composite nondifferentiable optimization problems. Over the past decades, many authors have studied the trust region algorithm to minimize nonsmooth objective function problems. For example, Sampaio, Yuan and Sun [23] used the trust region algorithm for nonsmooth optimization problems; Sun, Sampaio and Yuan [24] proposed a quasi-Newton trust region algorithm for nonsmooth least-squares problems; Zhang [25] used a new trust region algorithm for nonsmooth convex minimization; and Yuan, Wei and Wang [26] proposed a gradient trust region algorithm with a limited memory BFGS update for nonsmooth convex minimization problems. For other references on trust region methods, see [27–35], among others. In particular, for the problem we address in this study, as we can compute the exact Hessian, the trust region method could be very efficient. However, it is difficult to compute the Hessian at every iteration, which increases the computational workload and time.

The purpose of this paper is to present an efficient trust region algorithm to solve Eq (3). With the use of the Moreau-Yosida regularization (smoothing) and the new quasi-Newton equation, the given method has the following good properties: (i) the Hessian makes use of not only the gradient value but also the function value and (ii) the subproblem of the proposed method, which possesses the form of an unconstrained trust region subproblem, can be solved using existing methods.

The remainder of this paper is organized as follows. In the next section, we briefly review some basic results in convex analysis and nonsmooth analysis and state a new quasi-Newton secant equation. In section 3, we present a new algorithm for solving problem Eq (3). In section 4, we prove the global convergence of the proposed method. In section 5, we report numerical results and present some comparisons for the existing methods to solve problem Eq (1). We conclude our paper in Section 6.

Throughout this paper, unless otherwise specified, ‖ ⋅ ‖ denotes the Euclidean norm of vectors or matrices.

## Initial results

In this section, we first state some basic results in convex analysis and nonsmooth analysis. Let

$$\begin{array}{c} \theta \left(z,x\right)=f\left(z\right)+\frac{1}{2\lambda }{∥z-x∥}^{2}, \end{array}$$

and denote *p*(*x*): = *argmin*_*z* ∈ ℝ^*n*^_ *θ*(*z*, *x*). Then, *p*(*x*) is well defined and unique, as *θ*(*z*, *x*) is strongly convex. By Eq (2), *F* can be rewritten as

$$\begin{array}{c} F\left(x\right)=f\left(p\left(x\right)\right)+\frac{1}{2\lambda }{∥p(x)-x∥}^{2}. \end{array}$$

In the following, we denote *g*(*x*) = ∇*F*(*x*). Some important properties of *F* are given as follows:

1. *F* is finite-valued, convex and everywhere differentiable with
  $$\begin{array}{c} g\left(x\right)=\nabla F\left(x\right)=\frac{x-p(x)}{\lambda }. \end{array}$$ (4)
2. The gradient mapping *g* : ℝ^*n*^ → ℝ is globally Lipschitz continuous with modulus *λ*, i.e.,
  $$\begin{array}{c} ∥g\left(x\right)-g\left(y\right)∥\leq \frac{1}{\lambda }∥x-y∥,\;\;\forall x,y\in R^{n}. \end{array}$$ (5)
3. *x* solves Eq (1) if and only if ∇*F*(*x*) = 0, namely, *p*(*x*) = *x*.

It is obvious that *F*(*x*) and *g*(*x*) can be obtained through the optimal solution of *argmin*_*z* ∈ ℝ^*n*^_ *θ*(*z*, *x*). However, the minimizer of *θ*(*z*, *x*), *p*(*x*) is difficult or even impossible to solve for exactly. Thus, we cannot compute the exact value of *p*(*x*) to define *F*(*x*) and *g*(*x*). Fortunately, for each *x* ∈ ℝ^*n*^ and any *ϵ* > 0, there exists a vector *p* ^*α*^(*x*, *ϵ*) ∈ ℝ^*n*^ such that

$$\begin{array}{c} f(p^{\alpha }\left(x,\epsilon \right))+\frac{1}{2\lambda }{∥p^{\alpha }\left(x,\epsilon \right)-x∥}^{2}\leq F\left(x\right)+\epsilon . \end{array}$$ (6)

Thus, we can use *p* ^*α*^(*x*, *ϵ*) to define respective approximations of *F*(*x*) and *g*(*x*) as follows, when *ϵ* is small,

$$\begin{array}{c} F^{\alpha }\left(x,\epsilon \right):=f(p^{\alpha }\left(x,\epsilon \right))+\frac{1}{2\lambda }{∥p^{\alpha }\left(x,\epsilon \right)-x∥}^{2} \end{array}$$ (7)

and

$$\begin{array}{c} g^{\alpha }\left(x,\epsilon \right):=\frac{x-p^{\alpha }\left(x,\epsilon \right)}{\lambda }, \end{array}$$ (8)

The papers [36, 37] describe some algorithms to calculate *p* ^*α*^(*x*, *ϵ*). The following remarkable feature of *F* ^*α*^(*x*, *ϵ*) and *g* ^*α*^(*x*, *ϵ*) is obtained from [38].

**Proposition 2.1** Let *p* ^*α*^(*x*, *ϵ*) be a vector satisfying Eq (6), and *F* ^*α*^(*x*, *ϵ*) and *g* ^*α*^(*x*, *ϵ*) are defined by Eqs (7) and (8), respectively. Then, we obtain

$$\begin{array}{c} F\left(x\right)\leq F^{\alpha }\left(x,\epsilon \right)\leq F\left(x\right)+\epsilon , \end{array}$$ (9)

$$\begin{array}{c} ∥p^{\alpha }\left(x,\epsilon \right)-p\left(x\right)∥\leq \sqrt{2\lambda \epsilon }, \end{array}$$ (10)

and

$$\begin{array}{c} ∥g^{\alpha }\left(x,\epsilon \right)-g\left(x\right)∥\leq \sqrt{\frac{2\epsilon }{\lambda }}. \end{array}$$ (11)

The relations Eqs (9), (10) and (11) imply that *F* ^*α*^(*x*, *ϵ*) and *g* ^*α*^(*x*, *ϵ*) may be made arbitrarily close to *F*(*x*) and *g*(*x*), respectively, by choosing the parameter *ϵ* to be small enough.

Second, recall that when *f* is smooth, the quasi-Newton secant method is used to solve problem Eq (1). The iterate *x*_*k*_ satisfies ∇*f*_*k*_ + *B*_*k*_(*x*_*k*+1_ − *x*_*k*_) = 0, where ∇*f*_*k*_ = ∇*f*(*x*_*k*_), *B*_*k*_ is an approximation Hessian of *f* at *x*_*k*_, and the sequence of matrix {*B*_*k*_} satisfies the secant equation as follows.

$$\begin{array}{c} B_{k+1}s_{k}=y_{k}, \end{array}$$ (12)

where *y*_*k*_ = ∇*f*_*k*+1_− ∇*f*_*k*_ and *s*_*k*_ = *x*_*k*+1_ − *x*_*k*_. However, the function values are not exploited in Eq (12), which the method solves by only using the gradient information. Motivated by the above observations, we hope to develop a method that uses both the gradient information and function information. This problem has been studied by several authors. In particular, Wei, Li and Qi [39] proposed an important modified secant equation by using not only the gradient values but also the function values, and the modified secant is defined as

$$\begin{array}{c} B_{k+1}s_{k}=\nu_{k}, \end{array}$$ (13)

where *ν*_*k*_ = *y*_*k*_ + *β*_*k*_ *s*_*k*_, *f*_*k*_ = *f*(*x*_*k*_), ∇*f*_*k*_ = ∇*f*(*x*_*k*_), and $\beta_{k}=\frac{{\left(\nabla f_{k+1}+\nabla f_{k}\right)}^{T}s_{k}+2(f_{k}-f_{k+1})}{{\left\|s_{k}\right\|}^{2}}$. When *f* is twice continuously differentiable and *B*_*k*+1_ is updated by the BFGS formula [40–43], where *B*_*k*_ = *I* is a unit matrix if *k* = 0, this secant Eq (13) possesses the following remarkable property:

$$\begin{array}{c} f_{k}=f_{k+1}+\nabla f_{k+1}^{T}s_{k}+\frac{1}{2}s_{k}^{T}B_{k+1}s_{k} \end{array}$$

This property holds for all *k*. Based on the result of Theorem 2.1 [39], Eq (13) has an advantage over Eq (12) in this approximate relation.

## The new model

In this section, we present a modified BFGS formula using trust region model for solving Eq (1), which is motivated by the Moreau-Yosida regularization (smoothing), general trust region method and the new secant Eq (13). First, we describe the trust region method. In each iteration, a trial step *d*_*k*_ is generated by solving an adaptive trust region subproblem, in which the values of the gradient of *F*(*x*) at *x*_*k*_ and Eq (13) are used:

$$\begin{array}{ccc} & & \min\;q_{k}\left(d\right)=g^{\alpha }{\left(x_{k},\epsilon_{k}\right)}^{T}d+\frac{1}{2}d^{T}B_{k}d,\\ & & s.t.\;∥d∥\leq \Delta_{k}, \end{array}$$ (14)

where the scalar *ϵ*_*k*_ > 0 and Δ_*k*_ describe the trust region radius.

Let *d*_*k*_ be the optimal solution of Eq (14). The actual reduction is defined by

$$\begin{array}{c} Are\;d_{k}:=F^{\alpha }\left(x_{k},\epsilon_{k}\right)-F^{\alpha }\left(x_{k}+d_{k},\epsilon_{k+1}\right), \end{array}$$ (15)

and we define the predict reduction as

$$\begin{array}{c} Pre\;d_{k}:=-g^{\alpha }{\left(x_{k},\epsilon_{k}\right)}^{T}-\frac{1}{2}d_{k}^{T}B_{k}d_{k}. \end{array}$$ (16)

Then, we define *r*_*k*_ to be the ratio between *Are* *d*_*k*_ and *Pre* *d*_*k*_

$$\begin{array}{c} r_{k}:=\frac{Are\;d_{k}}{Pre\;d_{k}}. \end{array}$$ (17)

Based on the new secant Eq (13) and with *B*_*k*+1_ being updated by the BFGS formula, we propose a modified BFGS formula. The *B*_*k*+1_ is defined by

$$\begin{array}{c} B_{k+1}:=\{\begin{array}{cc} B_{k}, & \text{if}\;\;\;\;s_{k}^{T}\nu_{k}\leq 0,\\ B_{k}-\frac{B_{k}s_{k}s_{k}^{T}B_{k}}{s_{k}^{T}B_{k}s_{k}}+\frac{\nu_{k}{\nu_{k}}^{T}}{{\nu_{k}}^{T}s_{k}}, & \text{if}\;\;\;\;s_{k}^{T}\nu_{k}>0, \end{array} \end{array}$$ (18)

where *s*_*k*_ = *x*_*k*+1_ − *x*_*k*_, *y*_*k*_ = *g* ^*α*^(*x*_*k*+1_, *ϵ*_*k*+1_) − *g* ^*α*^(*x*_*k*_, *ϵ*_*k*_), *ν*_*k*_ = *y*_*k*_ + *β*_*k*_ *s*_*k*_ and

$$\begin{array}{c} \beta_{k}=\frac{\left(g^{\alpha }\left(x_{k+1},\epsilon_{k+1}\right)+g^{\alpha }{\left(x_{k},\epsilon_{k})\right)}^{T}s_{k}+2(F^{\alpha }\left(x_{k},\epsilon_{k}\right)-F^{\alpha }\left(x_{k+1},\epsilon_{k+1}\right)\right)}{{∥s_{k}∥}^{2}}, \end{array}$$

if *k* = 0, then *B*_*k*_ = *I*, and *I* is a unit matrix.

We now list the steps of the modified trust region algorithm as follows.

**Algorithm 1**.

**Step 0**. Choose *x*_0_ ∈ ℝ^*n*^, 0 < *σ*_1_ < *σ*_2_ < 1, 0 < *η*_1_ < 1 < *η*_2_, *λ* > 0, 0 ≤ *ɛ* ≪ 1, Δ_*max*_ ≥ Δ_0_ > 0 is called the maximum value of trust region radius, *B*_0_ = *I*, and *I* is the unit matrix. Let *k*: = 0.

**Step 1**. Choose a scalar *ϵ*_*k*+1_ satisfying 0 < *ϵ*_*k*+1_ < *ϵ*_*k*_, and calculate *p* ^*α*^(*x*_*k*_, *ϵ*_*k*_), $g^{\alpha }(x_{k},\epsilon_{k})=\frac{x_{k}-p^{\alpha }(x_{k},\epsilon_{k})}{\lambda }$. If *x*_*k*_ satisfies the termination criterion ‖*g* ^*α*^(*x*_*k*_, *ϵ*_*k*_)‖ ≤ *ɛ*, then stop. Otherwise, go to Step 2.

**Step 2**. *d*_*k*_ solves the trust region subproblem Eq (14).

**Step 3**. Compute *Are* *d*_*k*_, *Pre* *d*_*k*_, *r*_*k*_ using Eqs (15), (16) and (17).

**Step 4**. Regulate the trust region radius. Let

$$\begin{array}{c} \Delta_{k+1}:=\{\begin{array}{cc} \eta_{1}\Delta_{k}, & \text{if}\;\;\;\;r_{k}<\sigma_{1},\\ \Delta_{k}, & \text{if}\;\;\;\;\sigma_{1}\leq r_{k}\leq \sigma_{2},\\ \min\{\eta_{2}\Delta_{k},\Delta_{max}\}, & \text{if}\;\;\;\;r_{k}>\sigma_{2}. \end{array} \end{array}$$

**Step 5**. If the condition *r*_*k*_ ≥ *σ*_1_ holds, then let *x*_*k* + 1_ = *x*_*k*_ + *d*_*k*_, update *B*_*k* + 1_ by Eq (18), and let *k*: = *k* + 1; go back to Step 1. Otherwise, let *x*_*k*+1_: = *x*_*k*_ and *k*: = *k* + 1; return to Step 2.

Similar to Dennis and Moré [44] or Yuan and Sun [45], we have the following result.

**Lemma 1** *If and only if the condition* $s_{k}^{T}\nu_{k}>0$ *holds*, *B*_*k*+1_ *will inherit the positive property of* *B*_*k*_.

*Proof* “ ⇒ ” If *B*_*k*+1_ is symmetric and positive definite, then

$$\begin{array}{ccc} s_{k}^{T}B_{k+1}s_{k} & = & s_{k}^{T}[B_{k}-\frac{B_{k}s_{k}s_{k}^{T}B_{k}}{s_{k}^{T}B_{k}s_{k}}+\frac{\nu_{k}{\nu_{k}}^{T}}{{\nu_{k}}^{T}s_{k}}]s_{k}\\ & = & s_{k}^{T}B_{k}s_{k}-\frac{s_{k}^{T}B_{k}s_{k}s_{k}^{T}B_{k}s_{k}}{s_{k}^{T}B_{k}s_{k}}+\frac{s_{k}^{T}\nu_{k}{\nu_{k}}^{T}s_{k}}{{\nu_{k}}^{T}s_{k}}\\ & = & s_{k}^{T}\nu_{k}\\ & > & 0. \end{array}$$

“⇐” For the proof of the converse, suppose that $s_{k}^{T}\nu_{k}>0$ and *B*_*k*_ is symmetric and positive definite for all *k* ≥ 0. We shall prove that *x* ^*T*^ *B*_*k*+1_ *x* > 0 holds for arbitrary *x* ≠ 0 and *x* ∈ ℝ^*n*^ by induction. It is easy to see that *B*_0_ = *I* is symmetric and positive definite. Thus, we have

$$\begin{array}{ccc} x^{T}B_{k+1}x & = & x^{T}B_{k}x-\frac{x^{T}B_{k}s_{k}s_{k}^{T}B_{k}x}{s_{k}^{T}b_{k}s_{k}}+\frac{x^{T}\nu_{k}{\nu_{k}}^{T}x}{{\nu_{k}}^{T}s_{k}}\\ & = & x^{T}B_{k}x-\frac{{\left(x^{T}B_{k}s_{k}\right)}^{2}}{s_{k}^{T}B_{k}s_{k}}+\frac{{\left(x^{T}\nu_{k}\right)}^{2}}{{\nu_{k}}^{T}s_{k}}. \end{array}$$ (19)

Because *B*_*k*_ is symmetric and positive definite for all *k* ≥ 0, there exists a symmetric and positive definite matrix $B_{k}^{\frac{1}{2}}$ such that $B_{k}=B_{k}^{\frac{1}{2}}B_{k}^{\frac{1}{2}}$. Thus, by using the Cauchy-Schwartz inequality, we obtain

$$\begin{array}{ccc} {\left(x^{T}B_{k}s_{k}\right)}^{2} & = & {\left[x^{T}B_{k}^{\frac{1}{2}}B_{k}^{\frac{1}{2}}s_{k}\right]}^{2}={\left[{\left(B_{k}^{\frac{1}{2}}x\right)}^{T}\left(B_{k}^{\frac{1}{2}}s_{k}\right)\right]}^{2}\\ & \leq & {∥B_{k}^{\frac{1}{2}}x∥}^{2}∥B_{k}^{\frac{1}{2}}s_{k}∥\\ & = & {\left(B_{k}^{\frac{1}{2}}x\right)}^{T}\left(B_{k}^{\frac{1}{2}}x\right){\left(B_{k}^{\frac{1}{2}}s_{k}\right)}^{T}\left(B_{k}^{\frac{1}{2}}s_{k}\right)\\ & = & \left(x^{T}B_{k}x\right)\left(s_{k}^{T}B_{k}s_{k}\right). \end{array}$$ (20)

It is not difficult to prove that the above inequality holds true if and only if there exists a real number *γ*_*k*_ ≠ 0 such that $B_{k}^{\frac{1}{2}}x=\gamma_{k}B_{k}^{\frac{1}{2}}s_{k}$, namely, *x* = *γ*_*k*_ *s*_*k*_.

Hence, if Eq (20) strictly holds (and note that $s_{k}{\nu_{k}}^{T}>0$), then from Eq (19), we have

$$\begin{array}{ccc} x^{T}B_{k+1}x & > & x^{T}B_{k}x-\frac{{\left(x^{T}B_{k}s_{k}\right)}^{2}}{s_{k}^{T}B_{k}s_{k}}+\frac{{\left(x^{T}\nu_{k}\right)}^{2}}{{\nu_{k}}^{T}s_{k}}\\ & = & \frac{{\left(x^{T}\nu_{k}\right)}^{2}}{{\nu_{k}}^{T}s_{k}}>0. \end{array}$$

Otherwise, ${\left(x^{T}B_{k}s_{k}\right)}^{2}=(x^{T}B_{k}x)(s_{k}^{T}B_{k}s_{k})$; then, there exists *γ*_*k*_ such that *x* = *γ*_*k*_ *s*_*k*_. Thus,

$$\begin{array}{ccc} x^{T}B_{k+1}x & = & \frac{{\left[{\left(\gamma_{k}s_{k}\right)}^{T}\nu_{k}\right]}^{2}}{{\nu_{k}}^{T}s_{k}}\\ & = & \gamma_{k}^{2}s_{k}^{T}\nu_{k}>0. \end{array}$$

Therefore, for each 0 ≠ *x* ∈ ℝ^*n*^, we have *x* ^*T*^ *B*_*k*+1_ *x* > 0. This completes the proof.

Lemma 1 states that if $s_{k}^{T}\nu_{k}>0$, then the matrix sequence {*B*_*k*_} is symmetric and positive definite, which is updated by the BFGS formula of Eq (18).

## Convergence analysis

In this section, the global convergence of Algorithm 1 is established under the assumption that the following conditions are required.

**Assumption A**.

1. Let the level set Ω
  $$\begin{array}{c} \Omega =\{x\in R^{n}|F^{\alpha }\left(x,\epsilon \right)\leq F^{\alpha }\left(x_{0},\epsilon \right),\;\forall x_{0}\in R^{n}\}. \end{array}$$
2. *F* is bounded from below.
3. The matrix sequence {*B*_*k*_} is bounded on Ω, which means that there exists a positive constant *M* such that
  $$\begin{array}{c} ∥B_{k}∥\leq M\;\;\forall k. \end{array}$$
4. The sequence {*ϵ*_*k*_} converges to zero.

Now, we present the following lemma.

**Lemma 2** *If* *d*_*k*_ *is the solution of* Eq (14), *then*

$$\begin{array}{c} Pre\;d_{k}=q_{k}\left(0\right)-q_{k}\left(d_{k}\right)\geq \frac{1}{2}∥g^{\alpha }\left(x_{k},\epsilon_{k}\right)∥\min\{\Delta_{k},\frac{∥g^{\alpha }\left(x_{k},\epsilon_{k}\right)∥}{∥B_{k}∥}\}. \end{array}$$ (21)

*Proof* Similar to the proof of Lemma 7(6.2) in Ma [46]. Note that the matrix sequence {*B*_*k*_} is symmetric and positive definite; then, we present $d_{k}^{c}$ to be a Cauchy point at iteration point *x*_*k*_, which is defined by

$$\begin{array}{c} d_{k}^{c}=-\mu_{k}\frac{\Delta_{k}}{∥g^{\alpha }\left(x_{k},\epsilon_{k}\right)∥}g^{\alpha }\left(x_{k},\epsilon_{k}\right), \end{array}$$

where $\mu_{k}=\text{min}\{\frac{{\left\|g^{\alpha }(x_{k},\epsilon_{k})\right\|}^{3}}{\Delta_{k}{g^{\alpha }(x_{k},\epsilon_{k})}^{T}B_{k}g^{\alpha }(x_{k},\epsilon_{k})},1\}$. It is easy to verify that the Cauchy point is a feasible point, i.e., $\|d_{k}^{c}\|\leq \Delta_{k}$.

If $\frac{{\left\|g^{\alpha }(x_{k},\epsilon_{k})\right\|}^{3}}{\Delta_{k}{g^{\alpha }(x_{k},\epsilon_{k})}^{T}B_{k}g^{\alpha }(x_{k},\epsilon_{k})}>1$, then

$$\begin{array}{c} {∥g^{\alpha }\left(x_{k},\epsilon_{k}\right)∥}^{3}>\Delta_{k}{g^{\alpha }\left(x_{k},\epsilon_{k}\right)}^{T}B_{k}g^{\alpha }\left(x_{k},\epsilon_{k}\right), \end{array}$$

and

$$\begin{array}{c} d_{k}^{c}=-\frac{\Delta_{k}}{∥g^{\alpha }\left(x_{k},\epsilon_{k}\right)}g^{\alpha }\left(x_{k},\epsilon_{k}\right). \end{array}$$

Thus, we obtain

$$\begin{array}{ccc} Pre\;d_{k}^{c} & = & -q_{k}(-\frac{\Delta_{k}}{∥g^{\alpha }\left(x_{k},\epsilon_{k}\right)∥}g^{\alpha }\left(x_{k},\epsilon_{k}\right))\\ & = & -{g^{\alpha }\left(x_{k},\epsilon_{k}\right)}^{T}(-\frac{\Delta_{k}}{∥g^{\alpha }\left(x_{k},\epsilon_{k}\right)∥}g^{\alpha }\left(x_{k},\epsilon_{k}\right))\\ & & \;\;\;\;\;\;-\frac{1}{2}{\left(-\frac{\Delta_{k}}{∥g^{\alpha }\left(x_{k},\epsilon_{k}\right)∥}g^{\alpha }\left(x_{k},\epsilon_{k}\right)\right)}^{T}B_{k}(-\frac{\Delta_{k}}{∥g^{\alpha }\left(x_{k},\epsilon_{k}\right)∥}g^{\alpha }\left(x_{k},\epsilon_{k}\right))\\ & = & \frac{\Delta_{k}}{∥g^{\alpha }\left(x_{k},\epsilon_{k}\right)∥}{∥g^{\alpha }\left(x_{k},\epsilon_{k}\right)∥}^{2}-\frac{1}{2}\frac{\Delta_{k}^{2}}{{∥g^{\alpha }\left(x_{k},\epsilon_{k}\right)∥}^{2}}{g^{\alpha }\left(x_{k},\epsilon_{k}\right)}^{T}B_{k}g^{\alpha }\left(x_{k},\epsilon_{k}\right)\\ & \geq & \frac{1}{2}\Delta_{k}∥g^{\alpha }\left(x_{k},\epsilon_{k}\right)∥\\ & \geq & \frac{1}{2}∥g^{\alpha }\left(x_{k},\epsilon_{k}\right)∥\min\{\Delta_{k},\frac{∥g^{\alpha }\left(x_{k},\epsilon_{k}\right)∥}{∥B_{k}∥}\}. \end{array}$$

Otherwise, we have $d_{k}^{c}=-\frac{{\left\|g^{\alpha }(x_{k},\epsilon_{k})\right\|}^{2}}{{g^{\alpha }(x_{k},\epsilon_{k})}^{T}B_{k}g^{\alpha }(x_{k},\epsilon_{k})}g^{\alpha }(x_{k},\epsilon_{k})$. Thus, we obtain

$$\begin{array}{ccc} Pre\;d_{k}^{c} & = & -g^{\alpha }\left(x_{k},\epsilon_{k}\right)(-\frac{{∥g^{\alpha }\left(x_{k},\epsilon_{k}\right)∥}^{2}}{{g^{\alpha }\left(x_{k},\epsilon_{k}\right)}^{T}B_{k}g^{\alpha }\left(x_{k},\epsilon_{k}\right)}g^{\alpha }\left(x_{k},\epsilon_{k}\right))\\ & & \;\;\;\;\;\;-\frac{1}{2}{\left(-\frac{{∥g^{\alpha }\left(x_{k},\epsilon_{k}\right)∥}^{2}}{{g^{\alpha }\left(x_{k},\epsilon_{k}\right)}^{T}B_{k}g^{\alpha }\left(x_{k},\epsilon_{k}\right)}g^{\alpha }\left(x_{k},\epsilon_{k}\right)\right)}^{T}B_{k}(-\frac{{∥g^{\alpha }\left(x_{k},\epsilon_{k}\right)∥}^{2}}{{g^{\alpha }\left(x_{k},\epsilon_{k}\right)}^{T}B_{k}g^{\alpha }\left(x_{k},\epsilon_{k}\right)}g^{\alpha }\left(x_{k},\epsilon_{k}\right))\\ & = & \frac{1}{2}\frac{{∥g^{\alpha }\left(x_{k},\epsilon_{k}\right)∥}^{4}}{{g^{\alpha }\left(x_{k},\epsilon_{k}\right)}^{T}B_{k}g^{\alpha }\left(x_{k},\epsilon_{k}\right)}\\ & \geq & \frac{1}{2}\frac{{∥g^{\alpha }\left(x_{k},\epsilon_{k}\right)∥}^{2}}{∥B_{k}∥}\\ & \geq & \frac{1}{2}∥g^{\alpha }\left(x_{k},\epsilon_{k}\right)∥\min\{\Delta_{k},\frac{∥g^{\alpha }\left(x_{k},\epsilon_{k}\right)∥}{∥B_{k}∥}\}. \end{array}$$

Let *d*_*k*_ be the solution of Eq (14). Because $q_{k}(d_{k}^{c})\geq q_{k}(d_{k})$, we have

$$\begin{array}{c} Pre\;d_{k}=q_{k}\left(0\right)-q_{k}\left(d_{k}\right)\geq \frac{1}{2}∥g^{\alpha }\left(x_{k},\epsilon_{k}\right)∥\min\{\Delta_{k},\frac{∥g^{\alpha }\left(x_{k},\epsilon_{k}\right)∥}{∥B_{k}∥}\}. \end{array}$$

This completes the proof.

**Lemma 3** *Let Assumption A hold true and the sequence* {*x*_*k*_} *be generated by Algorithm 1. If* *d*_*k*_ *is the solution of* Eq (14), *then*

$$\begin{array}{c} |Are\;d_{k}-Pre\;d_{k}|=o\left({∥d_{k}∥}^{2}\right). \end{array}$$ (22)

*Proof* Let *d*_*k*_ be the solution of Eq (14). By using Taylor expansion, *F* ^*α*^(*x*_*k*_ + *d*_*k*_, *ϵ*_*k*+1_) can be expressed by

$$\begin{array}{c} F^{\alpha }\left(x_{k}+d_{k},\epsilon_{k+1}\right)=F^{\alpha }\left(x_{k},\epsilon_{k}\right)+{g^{\alpha }\left(x_{k},\epsilon_{k}\right)}^{T}d_{k}+\frac{1}{2}d_{k}^{T}B_{k}d_{k}+o\left({∥d_{k}∥}^{2}\right), \end{array}$$ (23)

Note that with the definitions of *Are* *d*_*k*_ and *Pre* *d*_*k*_ and by using Eq (23), we have

$$\begin{array}{ccc} |Are\;d_{k}-Pre\;d_{k}| & = & |F^{\alpha }\left(x_{k},\epsilon_{k}\right)-F^{\alpha }\left(x_{k}+d_{k},\epsilon_{k+1}\right)+q_{k}\left(d_{k}\right)|\\ & = & o({∥d_{k}∥}^{2}). \end{array}$$

The proof is complete.

**Lemma 4** *Let Assumption A hold. Then, Algorithm 1 does not circle in the inner cycle infinitely*.

*Proof* Suppose, by contradiction to the conclusion of the lemma, that Algorithm 1 cycles between Steps 2 and 5 infinitely at iteration point *x*_*k*_, i.e., *r*_*k*_ < *σ*_1_ and that there exists a scalar *ρ* > 0 such that ‖*g* ^*α*^(*x*_*k*_, *ϵ*_*k*_)‖ ≥ *ρ*. Thus, noting that 0 < *η*_1_ < 1, we have

$$\begin{array}{c} ∥d_{k}∥\leq \Delta_{k}=\eta_{1}^{k}\Delta_{0}\to 0,\;for\;k\to \infty . \end{array}$$

By using the result Eq (22) of Lemma 3 and the definition of *r*_*k*_, we obtain

$$\begin{array}{ccc} |r_{k}-1| & = & \frac{|Are\;d_{k}-Pre\;d_{k}|}{|Pre\;d_{k}|}\\ & \leq & \frac{2o({∥d_{k}∥}^{2})}{∥g^{\alpha }\left(x_{k},\epsilon_{k}\right)∥\min\{\Delta_{k},\frac{∥g^{\alpha }\left(x_{k},\epsilon_{k}\right)∥}{∥B_{k}∥}\}}\to 0,\;for\;k\to \infty . \end{array}$$

which means that we must have *r*_*k*_ ≥ *σ*_1_; this contradicts the assumption that *r*_*k*_ < *σ*_1_, and the proof is complete.

Based on the above lemmas, we can now demonstrate the global convergence of Algorithm 1 under suitable conditions.

**Theorem 1** *(Global Convergence). Suppose that Assumption A holds and that the sequence* {*x*_*k*_} *is generated by Algorithm 1. Let d_k_ be the solution of* Eq (14). *Then*, $\underset{k\to \infty }{\text{lim}}\text{inf}\|g_{k}\|=0$ *holds, and any accumulation point of x_k_ is an optimal solution of* Eq (1).

*Proof* We first prove that

$$\begin{array}{c} \lim_{k\to \infty }\inf∥g^{\alpha }\left(x_{k},\epsilon_{k}\right)∥=0. \end{array}$$ (24)

Suppose that *g* ^*α*^(*x*_*k*_, *ϵ*_*k*_) ≠ 0. Without loss of generality, by the definition of *r*_*k*_, we have

$$\begin{array}{c} |r_{k}-1|=|\frac{F^{\alpha }\left(x_{k}+d_{k},\epsilon_{k+1}\right)-F^{\alpha }\left(x_{k},\epsilon_{k}\right)-q_{k}\left(d_{k}\right)}{q_{k}\left(d_{k}\right)}|. \end{array}$$ (25)

Using Taylor expansion, we obtain

$$\begin{array}{c} F^{\alpha }\left(x_{k}+d_{k},\epsilon_{k+1}\right)=F^{\alpha }\left(x_{k},\epsilon_{k}\right)+{g^{\alpha }\left(x_{k},\epsilon_{k}\right)}^{T}d_{k}+\int_{0}^{1}d_{k}^{T}\left[g^{\alpha }\left(x_{k}+td_{k},\epsilon_{k+1}\right)-g^{\alpha }\left(x_{k},\epsilon_{k}\right)\right]dt. \end{array}$$

When Δ_*k*_ > 0 and small enough, we have

$$\begin{array}{ccc} & & \;|F^{\alpha }\left(x_{k}+d_{k},\epsilon_{k+1}\right)-F^{\alpha }\left(x_{k},\epsilon_{k}\right)-q_{k}\left(d_{k}\right)|\\ & = & \left|\frac{1}{2}d_{k}^{T}B_{k}d_{k}-\int_{0}^{1}d_{k}^{T}\left[g^{\alpha }\left(x_{k}+td_{k},\epsilon_{k+1}\right)-g^{\alpha }\left(x_{k},\epsilon_{k}\right)\right]dt\right|\\ & \leq & \frac{1}{2}M{∥d_{k}∥}^{2}+o(∥d_{k}∥). \end{array}$$ (26)

Suppose that there exists *ω*_0_ > 0 such that ‖*g* ^*α*^(*x*_*k*_, *ϵ*_*k*_)‖ ≥ *ω*_0_. By contradiction, using Eqs (25) and (26) and Lemma 2, we have

$$\begin{array}{ccc} |r_{k}-1| & \leq & \frac{\frac{1}{2}M{∥d_{k}∥}^{2}+o(∥d_{k}∥)}{\frac{1}{2}∥g^{\alpha }\left(x_{k},\epsilon_{k}\right)\min\{\Delta_{k},\frac{∥g^{\alpha }\left(x_{k},\epsilon_{k}\right)∥}{∥B_{k}∥}\}}\\ & \leq & \frac{M\Delta_{k}^{2}+o\left(\Delta_{k}\right)}{\omega_{0}\min\left\{\Delta_{k},\frac{\omega_{0}}{M}\right\}}\\ & = & O(\Delta_{k}). \end{array}$$ (27)

which means that there exists sufficiently small $\hat{\Delta }>0$ such that $\Delta_{k}\leq \hat{\Delta }$ for each *k*, and we have ∣*r*_*k*_ − 1∣ < 1 − *σ*_2_, i.e., *r*_*k*_ > *σ*_2_. Then, according to the Algorithm 1, we have Δ_*k*+1_ ≥ Δ_*k*_.

Thus, there exists a positive integer *k*_0_ and a constant *ρ*_0_ for arbitrary *k* ≥ *k*_0_ and satisfying $\Delta_{k}\leq \hat{\Delta }$, for which we have

$$\begin{array}{c} \Delta_{k}\neq \rho_{o}\hat{\Delta }. \end{array}$$ (28)

On the other hand, because *F* is bounded from below, and supposing that there exists an infinite number *k* such that *r*_*k*_ > *σ*_1_, by the definition of *r*_*k*_ and Lemma 2, for each *k* ≥ *k*_0_,

$$\begin{array}{ccc} & & \;\;\;\;\;\;F^{\alpha }\left(x_{k},\epsilon_{k}\right)-F^{\alpha }\left(x_{k}+d_{k},\epsilon_{k+1}\right)\\ & & >\sigma_{1}\left[q_{k}\left(0\right)-q_{k}\left(d_{k}\right)\right]\\ & & \geq \frac{\sigma }{2}\omega_{0}\min\{\Delta_{k},\frac{\omega_{0}}{M}\}. \end{array}$$

which means that Δ_*k*_ → 0 for *k* → ∞; this is a contradiction to Eq (28).

Moreover, suppose that for sufficiently large *k*, we have *r*_*k*_ < *σ*_1_. Then, $\Delta_{k}=\eta_{1}^{k}\Delta_{0}$, and we can see that Δ_*k*_ → 0 for *k* → ∞; this is also a contradiction to Eq (28). The contradiction shows that Eq (24) holds.

We now show that $\underset{k\to \infty }{\text{lim}}\text{inf}\|g_{k}\|=0$ holds. By using Eq (11), we have

$$\begin{array}{c} ∥g^{\alpha }\left(x_{k},\epsilon_{k}\right)-g\left(x_{k}\right)∥\leq \sqrt{\frac{2\epsilon_{k}}{\lambda }}. \end{array}$$

Together with Assumption A(iv), this implies that

$$\begin{array}{c} \lim_{k\to \infty }\inf∥g_{k}∥=0. \end{array}$$ (29)

Finally, we make a final assertion. Let x* be an accumulation point of {*x*_*k*_}. Then, without loss of generality, there exists a subsequence {*x*_*k*_}_*K*_ satisfying

$$\begin{array}{c} \lim_{k\to \infty ,k\in K}x_{k}=x^{*}. \end{array}$$ (30)

From the properties of *F*, we have

$$\begin{array}{c} g\left(x_{k}\right)=\frac{x_{k}-p\left(x_{k}\right)}{\lambda }. \end{array}$$

Thus, by using Eqs (29) and (30), we have x* = *p*(x*). Therefore, x* is an optimal solution of Eq (1). The proof is complete.

Similar to Theorem 3.7 in [25], we can show that the rate of convergence of Algorithm 1 is Q-superlinear. We omit this proof here (the proof of the Q-superlinear convergence can be found in [25]).

**Theorem 2** *(Q-superlinear Convergence)* [25] *Suppose that Assumption A(ii) holds, that the sequence* {*x*_*k*_} is generated by Algorithm 1, which has a limit point x*, and that g is BD-regular and semismooth at x*. Furthermore, suppose that *ϵ*_*k*_ = *o*(‖*g*(*x*_*k*_)‖^2^). *Then*,

1. x* is the unique solution of Eq (1);
2. *the entire sequence* {*x*_*k*_} converges to x* Q-superlinearly, i.e.,
  $$\begin{array}{c} \lim_{k\to \infty }\frac{∥x_{k+1}-x^{*}∥}{∥x_{k}-x^{*}∥}=0. \end{array}$$

## Results

In this section, we test our modified BFGS formula using a trust region model for solving nonsmooth problems. The type of nonsmooth problems addressed in Table 1 can be found in [47–53]. The problem dimensions and optimum function values are listed in Table 1, where “No.” is the number of the test problem, “Dim” is the dimension of the test problem, “Problem” is the name of the test problem, “*x*_0_” is the initial point, and “*f*_*ops*_(*x*)” is the optimization function evaluation. Here, the modified algorithm was implemented using MATLAB 7.0.4, and all numerical experiments were run on a PC with CPU Intel CORE(TM) 2 Duo T6600 2.20 GHZ, with 2.00 GB of RAM and with the Windows 7 operating system.

**Table 1:** Problem descriptions for test problems.

No.	Dim	Problem	*x*_0_	*f*_*ops*_(*x*)
1	2	Rosenbrock [47]	(-1.2, 1.0)	0
2	2	Crescent [47]	(-1.5, 2.0)	0
3	2	CB2 [48]	(1.0, -0.1)	1.9522245
4	2	CB3 [48]	(2.0, 2.0)	2.0
5	2	DEM [49]	(1.0, 1.0)	-3.0
6	2	QL [50]	(-1.0, 5.0)	7.20
7	2	LQ [50]	(-0.5, -0.5)	-1.4142136
8	2	Mifflin 2 [51]	(-1.0, -1.0)	-1.0
9	5	Shor [52]	(0.0, 0.0, 0.0, 0.0, 1.0)	22.600162
10	50	MXHILB [53]	ones(50, 1)	0
11	50	LIHILB [53]	ones(50, 1)	0

To test the performance of the given algorithm for the problems listed in Table 1, we compared our method with the trust region concept (BT) of paper [15], the proximal bundle method (PBL) of paper [17] and the gradient trust region algorithm with limited memory BFGS update (LGTR) described in [26]. The parameters were chosen as follows: *σ*_1_ = 0.45, *σ*_2_ = 0.75, *η*_1_ = 0.5, *η*_2_ = 4, *λ* = 1, Δ_0_ = 0.5 < Δ_*max*_ = 100 and $\epsilon_{k}=\frac{1}{{\left(2+k\right)}^{2}}$ (where *k* is the iterate number). We stopped the algorithm when the condition ‖*g* ^*α*^(*x*, *ϵ*)‖ ≤ 10^ − 6^ was satisfied. Based on the idea of [26], we use the function *fminsearch* in MATLAB for solving min *θ*(*z*, *x*). Then, we obtained the solution *p*(*x*); moreover, we obtained *g* ^*α*^(*x*, *ϵ*), which is computed using Eq (8). Meanwhile, we also listed the results of PBL, LGTR, BT and our modified algorithm in Table 2. The numerical results of PBL and BT can be found in [17], and the numerical results of LGTR can be found in [26]. The following notations are used in Table 2: “NI” is the number of iterations; “NF” is the number of the function evaluations; “*f*(*x*)” is the function value at final iteration; “——” indicates that the algorithm fails to solve the problem; and “Total” denotes the sum of the NI/NF.

**Table 2:** Test results.

No.	PBL NI/NF/*f*(*x*)	LGTR NI/NF/*f*(*x*)	BT NI/NF/*f*(*x*)	Algorithm 1 NI/NF/*f*(*x*)
1	42/45/3.81 × 10^−5^	——	79/88/1.30 × 10^−10^	26/66/4.247136 × 10^−6^
2	18/20/6.79 × 10^−5^	10/10/3.156719 × 10^−5^	24/27/9.44 × 10^−5^	13/13/2.521899 × 10^−5^
3	32/34/1.9522245	10/11/1.952225	13/16/1.952225	4/6/1.952262
4	14/16/2.0	2/3/2.000217	13/21/2.0	3/4/2.000040
5	17/19/-3.0	3/3/-2.999700	9/13/-3.0	4/24/-2.999922
6	13/15/7.2000015	19/119/7.200001	12/17/7.200009	9/9/7.200043
7	11/12/-1.4142136	1/1/-1.207068	10/11/-1.414214	2/2/-1.414214
8	66/68/-0.99999941	3/3/-0.9283527	6/13/-1.0	4/4/-0.9978547
9	27/29/22.600162	42/443/22.62826	29/30/22.600160	8/9/22.600470
10	19/20/4.24 × 10^−7^	12/12/9.793119 × 10^−3^	——	23/108/5.228012 × 10^−3^
11	19/20/9.90 × 10^−8^	20/63/9.661137 × 10^−3^	——	7/7/2.632534 × 10^−3^
Total	278/298	164/1111	353/412	103/252

**Fig 1:**
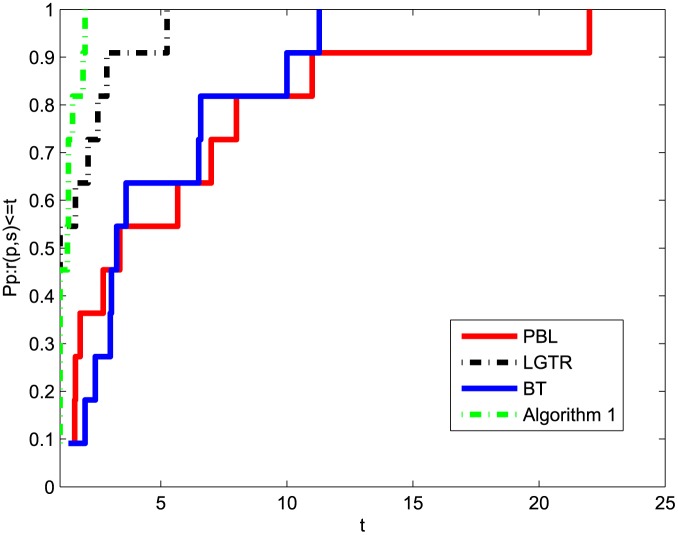
Performance profiles of these methods (NI).

**Fig 2:**
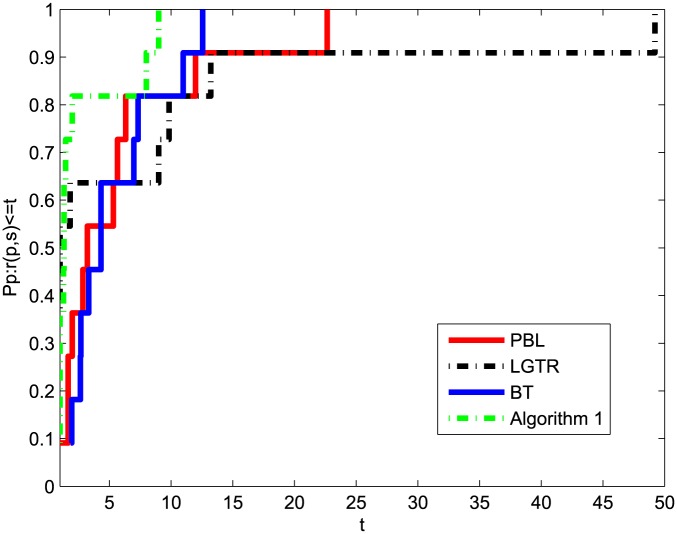
Performance profiles of these methods (NF).

The numerical results show that the performance of our algorithm is superior to those of the methods in Table 2. It can be seen clearly that the sum of our algorithm relative to NI and NF is less than the other three algorithms. The paper [54] provides a new tool for analyzing the efficiency of these four algorithms. Figs 1 and 2 show the performances of these four methods relative to NI and NF of Table 2, respectively. These two figures prove that Algorithm 1 provides a good performance for all the problems tested compared to PBL, LGTR and BT. In sum, the preliminary numerical results indicate that the modified method is efficient for solving nonsmooth convex minimizations.

## Conclusion

The trust region method is one of the most efficient optimization methods. In this paper, by using the Moreau-Yosida regularization (smoothing) and a new secant equation with the BFGS formula, we present a modified BFGS formula using a trust region model for solving nonsmooth convex minimizations. Our algorithm does not compute the Hessian of the objective function at every iteration, which decrease the computational workload and time, and it uses the function information and the gradient information. Under suitable conditions, global convergence is established, and we show that the rate of convergence of our algorithm is Q-superlinear. Numerical results show that this algorithm is efficient. We believe that this algorithm can be used in future applications to solve non smooth convex minimizations.
